# Multimodality imaging of a rare case of apical hypertrophic cardiomyopathy with endomyocardial fibrosis and myocardial calcification: case report and literature review

**DOI:** 10.3389/fcvm.2026.1774080

**Published:** 2026-02-18

**Authors:** Yuting Yi, Shuai Wang, Daoquan Peng

**Affiliations:** Department of Cardiovascular Medicine, the Second Xiangya Hospital of Central South University, Changsha, Hunan, China

**Keywords:** apical hypertrophic cardiomyopathy, endomyocardial fibrosis, multimodality imaging, myocardial calcification, PET-CT 18-fluorodeoxyglucose

## Abstract

Apical hypertrophic cardiomyopathy (ApHCM) complicated by endomyocardial fibrosis (EMF) and myocardial calcification (MC) is an extremely rare clinical entity. This report describes a 54-year-old woman with exertional dyspnea and edema. Multimodality imaging revealed apical hypertrophy with cavity obliteration, endocardial fibrosis, and calcification. Notably, ^18^F-FDG PET-CT showed intense apical uptake, initially raising suspicion for malignancy. This finding underscores the importance of recognizing that increased FDG uptake can occur in ApHCM to avoid false-positive oncologic interpretations. The diagnosis was confirmed by endomyocardial biopsy—consistent with hypertrophic cardiomyopathy—and genetic testing, which identified a pathogenic MYH7 variant (c.4145G>A, p.R1382Q). A systematic literature review identified 13 reported cases of ApHCM with calcification. All exhibited apical hypertrophy and calcification; most (10/13) had concurrent endomyocardial fibrosis. Genetic testing was performed in 7 cases, with positive results in only 4, highlighting the genetic heterogeneity of this phenotype. This case underscores the value of integrated multimodality imaging in delineating complex structural abnormalities, characterizing calcification, and differentiating this condition from malignancies. Accurate diagnosis requires a combination of clinical presentation, advanced imaging, histopathology, and genetic analysis to guide appropriate management and avoid misdiagnosis.

## 1 Introduction

Apical hypertrophic cardiomyopathy (ApHCM) is a phenotypic variant of hypertrophic cardiomyopathy characterized by localized thickening of the left ventricular apex (1). While its clinical and imaging features—such as the characteristic “ace of spades” configuration on echocardiography and the potential for apical cavity obliteration—are well documented in guidelines and recent literature (2–5), uncommon presentations pose diagnostic challenges. Among these, the coexistence of MC and intense ^18^F-fluorodeoxyglucose (^18^F-FDG) uptake in ApHCM is particularly rare and diagnostically complex.

MC, categorized as dystrophic or metastatic, is unusual in hypertrophic cardiomyopathy and may result from endomyocardial ischemia and subsequent fibrosis (6, 7). Its detection and characterization rely on multimodality imaging, including echocardiography, cardiac computed tomography (CT), and cardiac magnetic resonance (CMR) (6–10). Notably, ^18^F-FDG positron emission tomography-CT (PET-CT), used to identify malignant or inflammatory processes, may show focally increased apical uptake in ApHCM (11–18), mimicking cardiac neoplasms or inflammatory disorders such as sarcoidosis (19, 20).

This overlap creates a diagnostic dilemma: distinguishing ApHCM with calcification and metabolic hyperactivity from malignant or other infiltrative cardiac diseases. To date, only sporadic cases of ApHCM with calcification have been reported, and the role of integrated imaging in resolving this diagnostic uncertainty has not been comprehensively addressed. Therefore, this article presents a detailed case of ApHCM complicated by EMF and calcification, with intense apical ^18^F-FDG uptake, and provides a systematic review of published cases to summarize the clinical and imaging characteristics of this rare entity (18, 21–28). We aim to highlight the diagnostic challenges and the essential role of a multimodal imaging approach.

## 2 Case report

A 54-year-old woman presented with exertional dyspnea and edema. Her symptoms began 30 years prior with dyspnea during brisk walking or uphill climbing, but she did not seek medical evaluation. Three years before admission, exercise tolerance markedly declined, accompanied by mild bilateral lower limb edema that alleviated with diuretics. Over the preceding 20 days, edema progressed and was associated with orthopnea. Her medical history included an 8-year history of diabetes mellitus managed with metformin.

Physical examination revealed an irregular heart rhythm with a rate of 104 beats per minute and blood pressure of 118/84 mmHg. Jugular venous distension was present. Bilateral basilar crackles were heard and severe pitting edema was noted in both lower limbs.

Laboratory tests revealed elevated troponin T (22.16 pg/mL, Normal value < 14 pg/mL) and CK-MB (72.7 U/L, Normal value < 24 U/L), and NT-proBNP (3,572 pg/mL, Normal value < 125 pg/mL). Liver function tests showed elevated ALT (75.1 U/L, Normal value 9–50 U/L) and AST (48.2 U/L, Normal value 15–40 U/L), with decreased albumin (33.5 g/L, Normal value 40–55 U/L). Complete blood count (including eosinophil count), serum calcium, phosphorus, parathyroid hormone, renal function, erythrocyte sedimentation rate (ESR), C-reactive protein (CRP), antinuclear antibody (ANA), and antineutrophil cytoplasmic antibody (ANCA) were normal.

**Figure 1:**
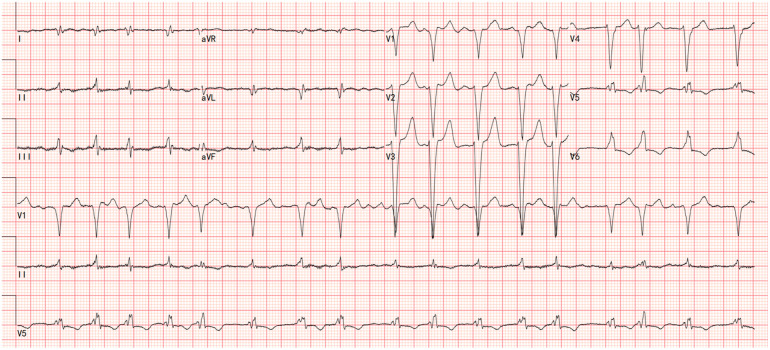
The electrocardiograms showed atrial fibrillation with a left bundle branch block (LBBB) and Q waves in leads I and aVL.

**Figure 2:**
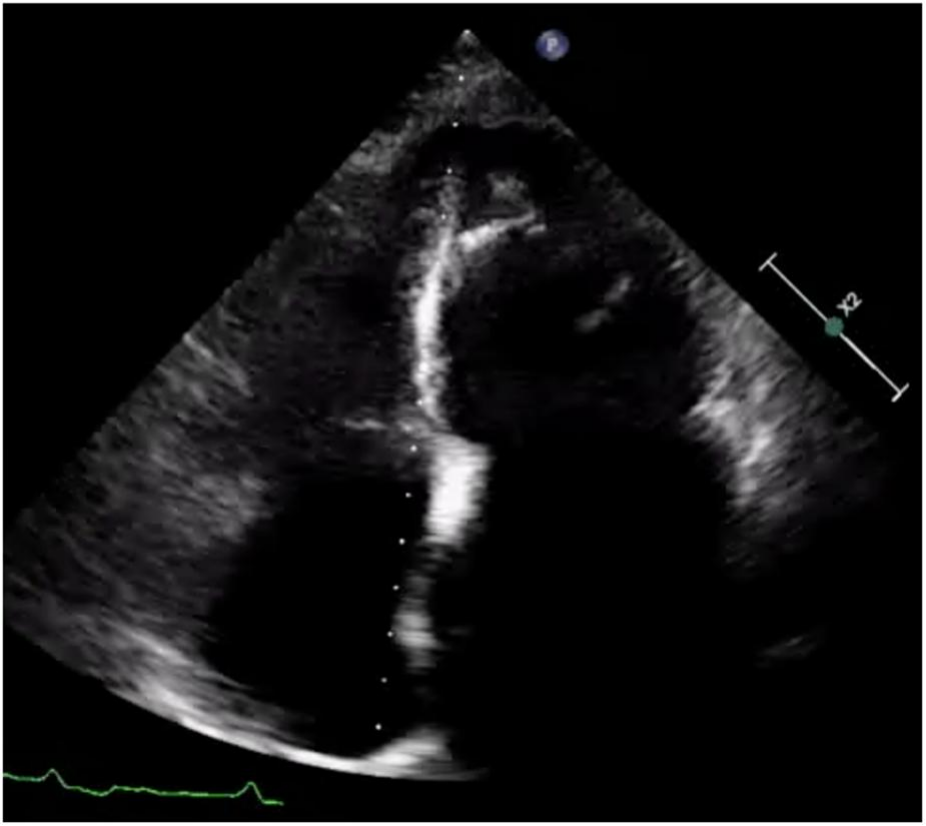
Apical 4-chamber view of the left ventricle showing apical hypertrophy in addition to asynergy in the apical walls with high echogenicity.

Electrocardiography showed atrial fibrillation with a left bundle branch block (LBBB) and Q waves in leads I and aVL (Figure 1). Transthoracic echocardiography demonstrated left ventricular and biatrial enlargement, with measured dimensions of a left ventricular end-diastolic diameter (LVDEd) 63 mm, left atrial systolic diameter (LAS) 58 mm, and right atrial diameter (RA) 43 mm. Additional findings included apical hypertrophy, increased endocardial echogenicity, diffusely hypokinetic wall motion, and a left ventricular ejection fraction (LVEF) of 40% (Figure 2). No left ventricular outflow tract or mid-cavity blood flow acceleration was detected.

**Figure 3:**
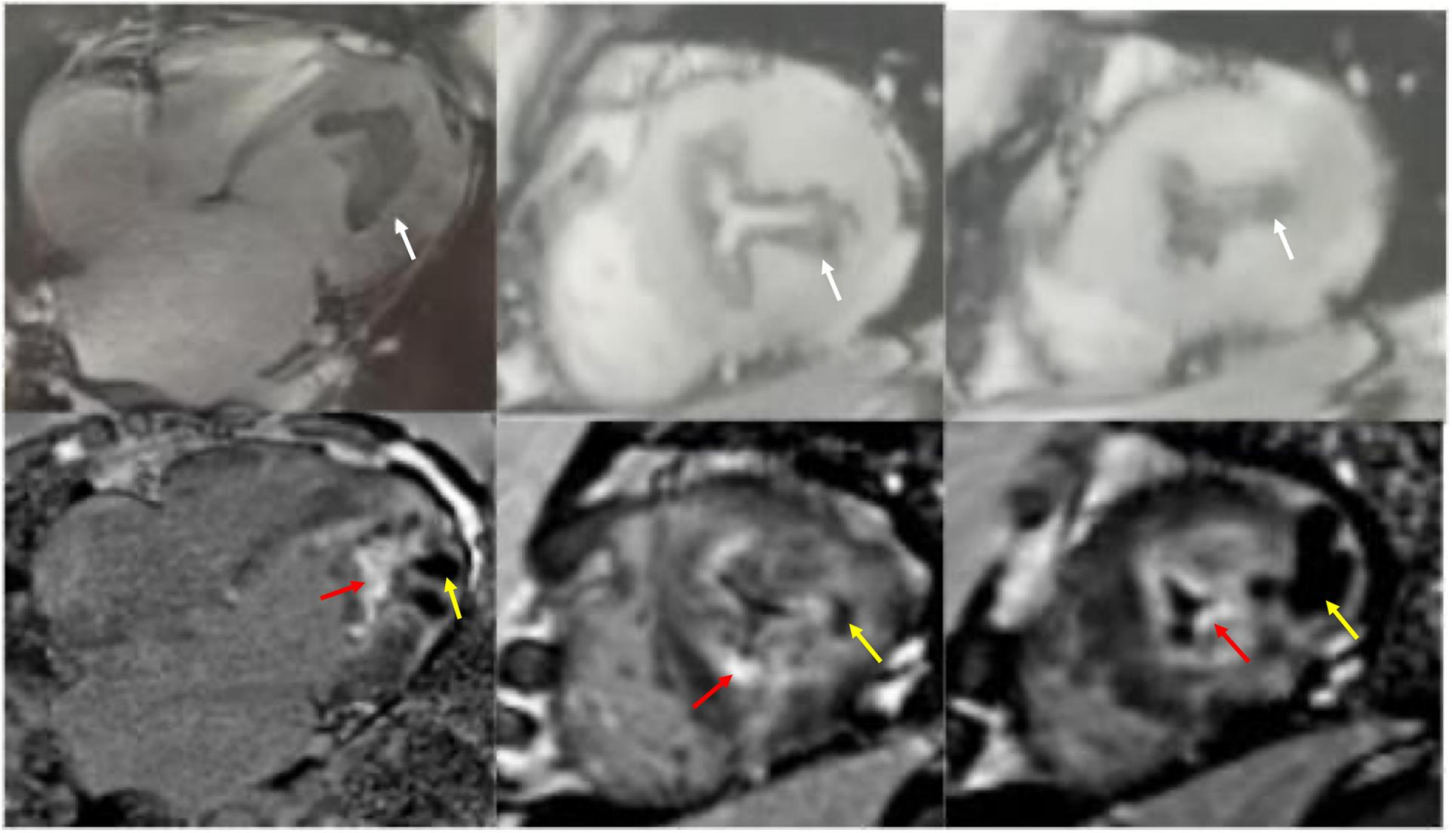
Cardiac magnetic resonance cine imaging revealed apical myocardial hypertrophy and a circumferential endocardial thrombus with low signal intensity, which showed no late gadolinium enhancement (white arrow). Nodular intra-myocardial lesions in the apical walls with marked hypokinesis were observed. These had a low signal in all sequences (yellow arrow). There was subendocardial and diffuse late gadolinium enhancement (red arrow).

**Figure 4:**
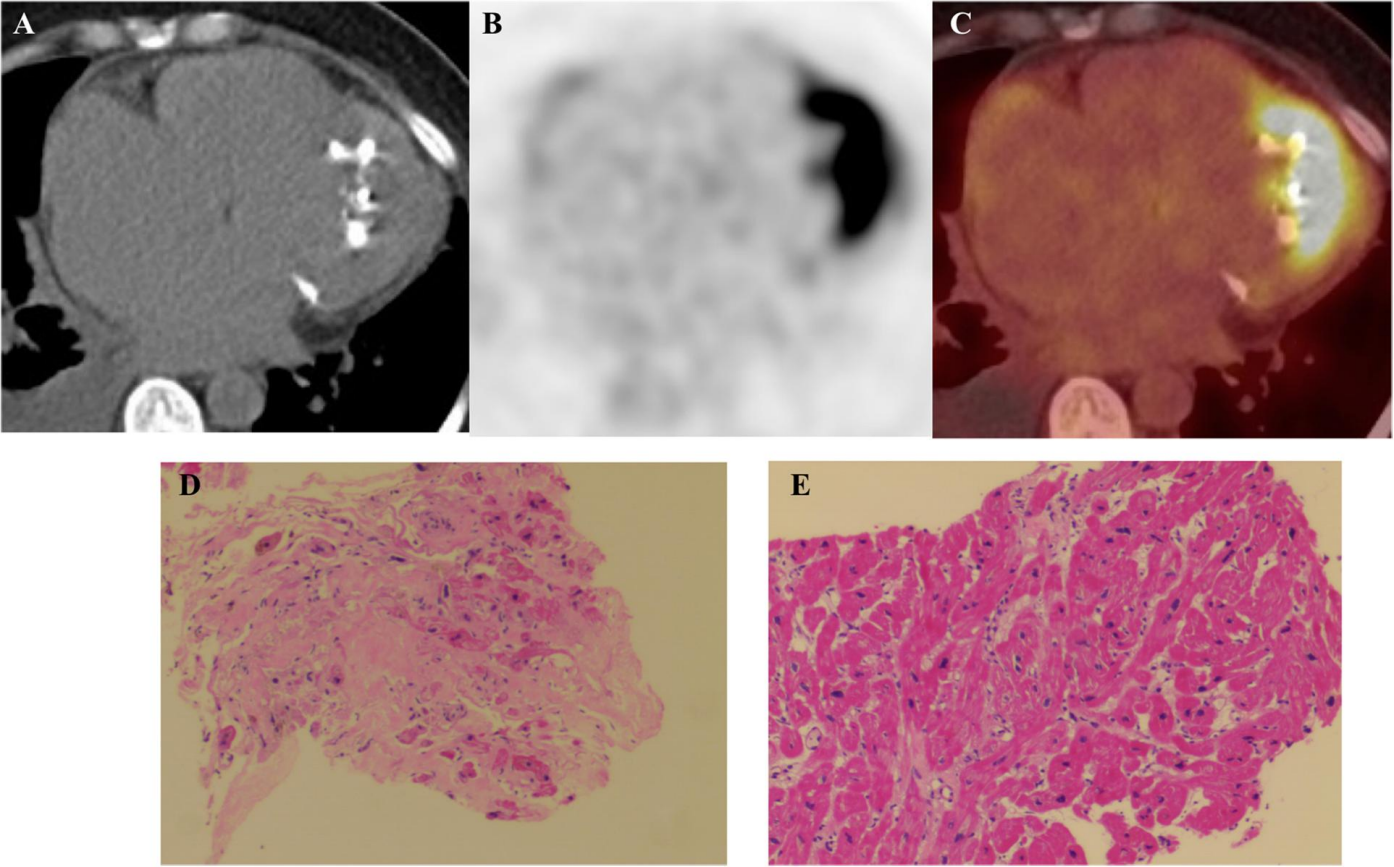
**(A)** Cardiac computed tomography shows marked calcification in the apex of the left ventricle (red arrow). **(B,C)** ^18^F-fluorodeoxyglucose (^18^F-FDG) positron emission tomography/computed tomography (PET/CT) fused images show avid ^18^F-FDG accumulation in the apical wall. **(D,E)** Myocardial Histopathology: Hematoxylin and eosin (H&E) staining of myocardial tissue (×100) reveals cardiomyocyte hypertrophy with focal atrophy, accompanied by lipofuscin deposition. The myocardial fibers show disarray, with focal interstitial fibrosis and minimal inflammatory cell infiltration.

CMR cine imaging confirmed apical myocardial hypertrophy. A circumferential endocardial region showed low signal intensity without late gadolinium enhancement (LGE), suggestive of thrombus. Within the myocardium, an area of low signal intensity on T1- and T2-weighted images showed no LGE. The LGE pattern was biphasic: one component showed marked subendocardial enhancement, while the other presented as diffuse, patchy enhancement within the myocardial wall (Figure 3). To exclude a cardiac tumor, ^18^F-FDG PET-CT was performed after an 18-hour fast, identifying MC and intense ^18^F-FDG uptake at the cardiac apex (Figures 4A–C). Thoracoabdominal CT showed no mass lesions or lymphadenopathy. Endomyocardial biopsy of the apical lesion under ultrasound guidance revealed hypertrophic and focally atrophic cardiomyocytes with lipofuscin deposition, myofiber disarray, and focal fibrosis—findings consistent with hypertrophic cardiomyopathy (Figures 4D,E). Integrated imaging and histopathological analysis revealed that the apical structure comprised endomyocardial fibrosis, dystrophic calcification, and minimal inflammatory infiltrates. Whole-exome sequencing identified a likely pathogenic MYH7 variant (NM_000257.4: c.4145G>A, p.R1382Q). The final diagnosis was ApHCM with EMF and MC.

The patient received metoprolol (47.5 mg daily), torasemide (10 mg daily), tolvaptan (7.5 mg daily), and rivaroxaban (15 mg daily). To date, cardiac myosin inhibitors have not been established to provide clinical benefit in non-obstructive HCM. Accordingly, this patient did not receive myosin inhibitors. Her paroxysmal nocturnal dyspnea resolved, and edema improved at discharge. However, during the 4-month follow-up, she had persistent exertional dyspnea and edema, with a New York Heart Association (NYHA) functional class III. Four months after discharge, she was readmitted due to heart failure exacerbation from a respiratory tract infection, with aggravated edema and abdominal distention requiring intravenous diuretics. NT-proBNP was elevated (6,420 pg/mL). Echocardiography showed a left ventricular internal diameter of 57 mm and LVEF of 35%.

## 3 Systematic literature review

A systematic literature search was conducted in PubMed database using keywords “apical hypertrophic cardiomyopathy” AND “calcification”, “myocardial calcification” AND “hypertrophic cardiomyopathy", "endomyocardial fibrosis” AND “ hypertrophic cardiomyopathy” from the database's inception to 2025. The inclusion criteria were restricted to case reports or case series describing confirmed ApHCM with MC. Studies were excluded if they contained insufficient data, involved non-human subjects, or were duplicate publications. Relevant data on demographics, clinical manifestations, imaging features, diagnostic methods, treatment, and prognosis were extracted. Case reports quality was assessed using the CARE checklist.

Thirteen cases of ApHCM with MC have been reported (Table 1). Among these, six patients were female and seven male, with a median age of 60 years (range 29–88). Echocardiography, performed in all 13 patients, detected apical hypertrophy, hypokinesis of the apical segment, and mural thrombus. CMR, performed in 11/13 patients, was central for diagnosis and differential diagnosis, characterizing fibrosis, calcification, and the typical “double V” sign. CT, employed in 9/13 patients, best delineated calcification extent and nature. Radionuclide imaging (SPECT/PET) was used less frequently for perfusion and metabolism assessment. All patients (13/13) exhibited both apical hypertrophy and calcification; most (10/13) had concurrent endomyocardial fibrosis. Complications included thrombus formation (3 patients) and left ventricular dilation or aneurysm (3 patients). Genetic testing was performed in 7 patients, with mutations identified in 4 (involving MYH7, MYBPC3, TMPO, and GATA6). The remaining 3 were negative, underscoring the limited genetic detection rate and heterogeneity in this ApHCM phenotype (18, 21–28).

**Table 1:** Reported cases of apical hypertrophic cardiomyopathy with calcification: a summary of case characteristics.

Literature (year)	Age/Sex	Clinical presentation	Imaging findings	Calcification features	Diagnostic confirmation	Treatment & follow-up
Park et al. (2007) (18)	29, Male	Abnormal CXR on routine exam; asymptomatic.	X-ray/Angio: Round apical calcification, "hour-glass” LV cavity with apical aneurysm. PET/CT: Apical hypertrophy, crescent-shaped calcified aneurysm with no FDG uptake (non-viable). CTA: Detailed 3D anatomy of the aneurysm.	Crescent-shaped calcification of the apical aneurysm wall, related to chronic ischemia and necrosis.	Integrated PET/CT confirmed metabolically inert, calcified aneurysm. Supported chronic ischemic injury pathogenesis.	Anticoagulation recommended for thromboembolism prevention.
Kaimoto et al. (2012) (24)	72, Male	ECG abnormality; asymptomatic.	Echo: Apical hypertrophy with hyperechoic apical mass, initially suspected as thrombus. CT: Confirmed intramyocardial calcification at LV apex, no coronary stenosis. No change with anticoagulation.	Intramyocardial calcification within the hypertrophied apex, without thrombus or aneurysm.	CT definitively characterized the finding as intramyocardial calcification, not thrombus. Calcification increased slightly over years.	Anticoagulation discontinued. Stable for >2 years without therapy.
Ito et al. (2015) (23)	88, Male	Incidental LV asynergy on pre-op screening; asymptomatic.	Echo: Apical hypertrophy (21 mm), asynergy with high echogenicity in posterolateral wall & apex. CT/CMR: Severe LV myocardial calcification (predominantly apical), normal coronaries. LGE showed enhancement in hypertrophied apex with hypointense endocardial layer (calcification).	Extensive intramyocardial (dystrophic) calcification, possibly related to remote rheumatic fever.	CT demonstrated extensive myocardial calcification. Clinical history supported dystrophic etiology.	Stress echo revealed latent risk of HF (increased TR gradient on exercise). Managed perioperatively as restrictive cardiomyopathy.
Muthukumar et al. (2016) (25)	48, Male	Chronic dyspnea	Echo: Apical hypertrophy (21 mm), echolucent apical mass with dense rim. CMR: Apical obliteration, endomyocardial calcification. LGE showed two distinct patterns: bright subendocardial hyperenhancement (consistent with EMF) and diffuse intramyocardial patchy hyperenhancement (consistent with ApHCM). CT: Marked apical calcification, no coronary stenosis.	Endomyocardial calcification associated with EMF.	Multimodality imaging (Echo, CMR, CT) suggested coexistent ApHCM and EMF. Genetic testing negative.	ICD implanted for primary SCD prevention.
Saba et al. (2017) (27)	76, Female	AF, chest pain, dyspnea, prior syncope.	CT: Circumferential apical myocardial calcification, mild apical hypertrophy, partial systolic cavity obliteration, dyskinesis. CCTA: Only minimal non-calcified plaque. Echo: Calcification less conspicuous.	Circumferential apical myocardial calcification, attributed to chronic microvascular ischemia.	CT was optimal for identifying calcification. Absence of significant CAD argued against atherosclerotic infarction.	Case report; specific management not detailed.
Huang et al. (2019) (22)	Case 1: 61, F Case 2: 60, F	Atypical chest pain, dyspnea, exercise intolerance, palpitations.	Echo: Apical hypertrophy, EMF with calcification, LA & LV enlargement (EF >55%). CMR/SPECT: Confirmed apical hypertrophy and ischemia.	Endomyocardial fibrosis with calcification	Multimodality imaging diagnosis of ApHCM with secondary EMF/calcification. Genetic testing negative for HCM (one had DCM-associated TMPO mutation).	Treated with ACEI, metoprolol, aspirin. No improvement on serial imaging, suggesting potentially poor prognosis.
Sehly et al. (2022) (28)	68, Male	Initially asymptomatic. Presented 2 years later with mild cognitive impairment, slurred speech, gait disturbance (cerebral infarcts).	Echo (initial): Biventricular apical obliteration, no thrombus. Favored ApHCM. CT (2 yrs later): Hypodense apical filling defects (thrombi) in LV & RV, apical hypertrophy (13 mm). CMR (post-treatment): LV apical thickening (15 mm). LGE showed two patterns: subendocardial “V” sign (EMF) and focal patchy mid-myocardial enhancement (ApHCM).	Focus on distinguishing EMF from ApHCM; calcification not a primary feature.	CMR LGE patterns were diagnostic for co-existing ApHCM and EMF. Peripheral eosinophilia supported active EMF phase.	Initially managed expectantly for ApHCM. After embolic events, started on high-dose oral steroids and anticoagulation for EMF with apical thrombi. Serial echo showed regression in apical thickness.
Gao et al. (2023) (21)	57, Female	Chest tightness, palpitations, shortness of breath, fatigue (worsening over 3 years).	Echo/Contrast Echo: Apical hypertrophy (20 mm), endocardial calcification, LV thrombus, ventricular septal aneurysm, “apple-shaped” LV cavity. CT/CMR: Apical calcification & hypertrophy. LGE showed subendocardial arc-shaped hyperenhancement (fibrosis) and endocardial hypointensity (calcification) – “double V” sign.	Apical endocardial calcification, potentially related to EMF, prior myocarditis, or organized thrombus.	CMR demonstrated classic EMF "double V” sign. Genetic testing revealed missense mutations in MYH7 and GATA6 genes.	Treated with ARNI, *β*-blocker, diuretics, SGLT2i. Rivaroxaban initiated for LV thrombus and TIA. Symptoms improved with normal daily activities.
Radano et al. (2024) (26)	Case Series (5 pts): 48F, 41M, 50F, 71M, 65M	Mostly asymptomatic (NYHA I); one with mild dyspnea (NYHA II).	Echo: All showed apical hypertrophy, obliteration, “ace-of-spades” sign, and fibrocalcific material on endocardial side. CMR: Universal pattern: hypointense component on EGE (calcium) + subendocardial LGE (fibrosis), suggesting EMF	Intramyocardial calcification with fibrosis, a rare finding in ApHCM. All patients had comorbid inflammatory triggers (obesity, malaria, thalassemia, microangiopathy, CKD/DM).	Characteristic CMR pattern (calcification on EGE + subendocardial LGE) was key for diagnosing concomitant EMF.	Based on ESC SCD risk score, only one high-risk patient received an ICD. Others continued on β-blockers/CCBs & diuretics with 6-month follow-up.

## 4 Discussion

ApHCM with EMF and calcification is an extremely rare, with only 13 reported worldwide (18, 21–28).

### 4.1 Pathogenesis of myocardial calcification

MC is categorized as dystrophic or metastatic: Dystrophic calcification occurs in necrotic or degenerated tissues, such as those affected by myocardial infarction, endomyocardial fibrosis, myocarditis, cardiac tumors, or radiation injury. Metastatic calcification is associated with calcium-phosphate metabolism disturbances, as in chronic renal failure or hyperthyroidism (6, 8). Hypertrophic cardiomyopathy is a rare cause of dystrophic calcification (6), with its mechanism linked to EMF secondary to myocardial ischemia (26). Apical hypertrophy is often accompanied by microvascular ischemia; dynamic small vessels obstruction from cavity obliteration and sustained apical contraction during diastole further aggravates subendocardial ischemia (29). This process promotes endomyocardial fibrosis, which may lead to dystrophic calcification (26).

### 4.2 Diagnostic value of multimodality imaging

In this case, multimodality imaging combined with biopsy and genetic testing was decisive for diagnosis. Although the patient lacked typical ApHCM ECG findings (giant inverted T waves) and maximum apical wall thickness was only 13 mm (below the ≥ 15 mm diagnostic threshold (3), multiple imaging modalities consistently revealed characteristic apical morphological abnormalities and cavity obliteration. The uniform wall-thickness cutoff may overlook physiological tapering, potentially underdiagnosing early or mild apical hypertrophy (3, 4, 30). Recent studies have reported that CMR-based strain imaging, particularly global longitudinal strain (GLS) and regional strain analysis, provides a quantitative assessment of myocardial deformation that can aid in differentiating ApHCM from other causes of ventricular hypertrophy, such as cardiac amyloidosis and hypertensive heart disease (31, 32).

For evaluating MC, echocardiography is the most commonly used, showing hyperechoic lesions with acoustic shadowing, though its diagnostic accuracy limited by poor acoustic windows (6). Cardiac CT is the gold standard, showing increased density in Hounsfield units (6, 8). CMR depicts calcification as low signal intensity on T1- and T2-weighted sequences without LGE in calcified regions (9, 10, 33). CMR also evaluates concomitant EMF and mural thrombus.

When interpreting apical LGE, it is crucial to distinguish it from slow-flow artifact, a known phenomenon in ApHCM with cavity obliteration (34). However, CMR diagnosis mandates that LGE must not be interpreted in isolation but rather through the integrative analysis of all CMR sequences and multimodality findings (35). In this case, the apical calcification on CT, characteristic signal changes across CMR sequences (e.g., signal voids on T1- and T2-weighted images), and the correlation with the LGE pattern collectively supported a fibrocalcific substrate with calcification rather than mere stagnant blood.

### 4.3 Metabolic features and differential significance of ^18^F-FDG PET-CT

In this case, ^18^F-FDG PET-CT showed focal apical hypermetabolism, mimicking malignancy. Under normal fasting conditions, myocardial FDG uptake is generally low was energy demands are met via fatty acid metabolism (36). Normal variations include lower uptake in the septum and anterior wall and higher uptake in lateral and posterior walls; basal ring-like or focal uptake is also common (36). Focal increased FDG avidity occurs in cardiac tumors and sarcoidosis (20). Patients with ApHCM may exhibit abnormally increased apical ^18^F-FDG uptake (12, 15–17), reflecting enhanced glucose utilization and impaired fatty acid metabolism due to microvascular ischemia and possible inflammation (37).

### 4.4 Differential diagnosis

The imaging presentation of ApHCM with calcification and intense FDG uptake necessitates differentiation from several other cardiac conditions. Key entities and their distinguishing multimodality imaging features are summarized below.

#### 4.4.1 Cardiac sarcoidosis vs. primary/metastatic cardiac neoplasms

Both cardiac sarcoidosis and cardiac tumors may present with focal wall thickening, abnormal metabolism on ^18^F-FDG PET-CT, and late gadolinium enhancement (LGE) on CMR, mimicking a neoplastic or inflammatory process.

#### 4.4.2 Cardiac sarcoidosis

CMR is pivotal, typically showing LGE in a non-ischemic, mid-myocardial or epicardial pattern, often involving the basal septum and lateral wall. Concomitant mediastinal or hilar lymphadenopathy on CT or PET supports the diagnosis. Myocardial FDG uptake usually shows a focal or patchy pattern corresponding to active inflammation.

#### 4.4.3 Cardiac tumors (primary/metastatic)

Echocardiography serves as the initial screening tool for identifying intracavitary or intramural masses. CMR aids in tissue characterization; malignancies often exhibit heterogeneous LGE and may show invasion into adjacent structures. On ^18^F-FDG PET-CT, most malignant tumors demonstrate intense, focal hypermetabolism. The absence of the characteristic apical “spade-shaped” hypertrophy and cavity obliteration of ApHCM, along with a more mass-like morphology, are distinguishing features.

#### 4.4.4 Endomyocardial fibroelastosis (EFE)

EFE is a restrictive cardiomyopathy characterized by diffuse endocardial thickening. Echocardiography is diagnostic, showing diffuse, markedly hyperechoic endocardial thickeningleading to ventricular cavity restriction or obliteration, often with preserved wall thickness but severely impaired systolic function. CMR confirms this, displaying the thickened endocardium as a diffuse, low-signal layer on both T1- and T2-weighted images with linear subendocardial LGE. Unlike ApHCM, myocardial hypertrophy is not a typical feature of EFE.

#### 4.4.5 Distinguishing left ventricular apical thrombus from calcification

This distinction is crucial in ApHCM, where both complications can occur.

Thrombus typically appears as an intracavitary mass adherent to akinetic or dyskinetic myocardium (e.g., within an apical aneurysm). On CMR, thrombus shows no enhancement on first-pass perfusion or LGE sequences. On cardiac CT, it appears as a low- or intermediate-attenuation filling defect without contrast enhancement.

Calcification presents as intramural or endocardial hyperdense lesions. Cardiac CT is the gold standard, showing very high attenuation (HU >130). On CMR, calcification appears as a signal void (very low signal) on all sequences, including cine, T1-, T2-weighted, and LGE. This persistent signal void across all CMR sequences reliably differentiates calcification from thrombus or enhancing tissue.

In summary, an integrated assessment using echocardiography for structure and function, CMR for tissue characterization (especially LGE patterns and signal voids), CT for definitive calcification detection, and PET-CT for metabolic activity is essential to navigate this complex differential diagnosis and avoid misdiagnosis.

### 4.5 Study limitations

This study has several limitations. First, as a case report and literature review, it is descriptive and retrospective in nature. Second, the analysis is based on a single case report and a synthesis of 13 published cases, limiting generalizability and statistical power. Third, follow-up was short (4 months). Fourth, genetic testing revealed significant heterogeneity and a low positivity rate; the clinical significance of some mutations remains uncertain, limiting genotype-phenotype correlations. Finally, the study focused on imaging and diagnosis, not systematic analysis of treatment efficicacy or prognostic factors.

## 5 Conclusion

In summary, ApHCM with MC is rare, its development is closely associated with the pathological cascade of endocardial ischemia, fibrosis, and calcification. Integrated multimodality imaging is essential not only for diagnosis rare calcification variants of ApHCM but also for preventing misdiagnosis in cases with atypical PET metabolic patterns.

## Data Availability

The original contributions presented in the study are included in the article/Supplementary Material, further inquiries can be directed to the corresponding authors.
