# Supplementary material for: Multimodality imaging of a rare case of apical hypertrophic cardiomyopathy with endomyocardial fibrosis and myocardial calcification: case report and literature review
Source: Front Cardiovasc Med. 2026 Feb 18;13:1774080. doi: 10.3389/fcvm.2026.1774080 (PMC12956803; doi:10.3389/fcvm.2026.1774080)
Supplement: Supplementary file 1 [file Datasheet1.docx]

**2013 CARE Checklist**

**1. Title – The diagnosis or intervention of primary focus followed by the words “case report”.**

**Adherent.** Title ends with “: Case Report and Literature Review”.

**2. Key Words** – 2 to 5 key words that identify diagnoses or interventions in this case report (including "case report").

**Adherent.** Key Word with “: Apical hypertrophic cardiomyopathy, Myocardial calcification, Multimodality imaging, Endomyocardial fibrosis”.

**3. Abstract** – (structured or unstructured)

- Introduction – What is unique about this case and what does it add to the scientific literature?
- The patient’s main concerns and important clinical findings.
- The primary diagnoses, interventions, and outcomes.
- Conclusion – What are one or more “take-away” lessons from this case report?

**Adherent (Unstructured).** **Uniqueness** (extremely rare entity, FDG uptake mimicking neoplasm). **Concerns & findings** (dyspnea, edema; imaging findings). **Diagnosis, Intervention, Outcome** (Confirmed by biopsy/genetics; pharmacotherapy; symptom partially improved). **Conclusion/Take-away** (Role of integrated imaging, holistic approach to avoid misdiagnosis).

**4. Introduction** – Briefly summarizes why this case is unique and may include medical literature references.

**Adherent.** Last paragraph clearly states the purpose: to report a rare case of ApHCM with fibrosis/calcification, intense FDG uptake requiring differential diagnosis, and to provide a systematic review.

**5. Patient Information**

- De-identified patient specific information.
- Primary concerns and symptoms of the patient.
- Medical, family, and psychosocial history including relevant genetic information.
- Relevant past interventions and their outcomes.

**Adherent.** Case Report Section: (1) 54-year-old female. (2) Exertional dyspnea, edema, orthopnea. (3) 8-year history of diabetes; genetic info (MYH7 variant) in *Diagnostic Assessment*. (4) Past diuretic therapy alleviated mild edema.

**6. Clinical Findings** – Describe significant physical examination (PE) and important clinical findings.

**Adherent.** Case Report Section: Irregular heart rhythm, jugular venous distension, bilateral crackles, severe pitting edema.

**7. Timeline** – Historical and current information from this episode of care organized as a timeline (figure or table).

| Time Point | Clinical Events and Diagnostic Interventions |
| --- | --- |
| **Symptom Onset (Approximately 30 years prior to admission)** | Exertional dyspnea (during brisk walking or uphill climbing). No medical evaluation sought. |
| **3 years prior to admission** | Marked decline in exercise tolerance, accompanied by mild bilateral lower limb edema. Edema alleviated with diuretic therapy. |
| **20 days prior to admission** | Progressive worsening of edema, associated with orthopnea. |
| **At admission (Day 0)** | Hospitalized for “exertional dyspnea and edema.” Physical examination, laboratory tests (notably elevated troponin T and NT-proBNP), electrocardiography (atrial fibrillation with LBBB), and transthoracic echocardiography (apical hypertrophy, chamber enlargement, LVEF 40%) were performed. |
| **During hospitalization** | Advanced multimodality imaging: Cardiac magnetic resonance (CMR), ¹⁸F-FDG PET-CT, thoracoabdominal CT. Endomyocardial biopsy under ultrasound guidance and whole-exome sequencing were completed. |
| **Upon diagnostic confirmation** | Final diagnosis established: Apical hypertrophic cardiomyopathy with endomyocardial fibrosis and myocardial calcification, confirmed by imaging, histopathology, and identification of a pathogenic MYH7 variant. |
| **At treatment initiation** | Combined pharmacotherapy initiated: metoprolol, torasemide, tolvaptan, and rivaroxaban. |
| **At discharge** | Paroxysmal nocturnal dyspnea resolved. Edema improved compared to admission. The patient was discharged. |
| **3-month follow-up after discharge** | The patient continued to exhibit exertional dyspnea and persistent edema, with a New York Heart Association (NYHA) functional class III. |

**8. Diagnostic Assessment**

- Diagnostic methods (PE, laboratory testing, imaging, surveys).
- Diagnostic challenges.
- Diagnosis (including other diagnoses considered).
- Prognostic characteristics when applicable.

**Adherent.** Scattered across Case Report & Discussion: (1) ECG, Echo, CMR, PET-CT, CT, Biopsy, Genetic test. (2) Atypical ECG, wall thickness <15mm, FDG uptake mimicking tumor (explicitly discussed). (3) Final diagnosis clearly stated. Cardiac tumor was the key differential. (4) NYHA class III at 3-month follow-up indicates poor prognosis.

**9. Therapeutic Intervention**

- Types of therapeutic intervention (pharmacologic, surgical, preventive).
- Administration of therapeutic intervention (dosage, strength, duration).
- Changes in therapeutic interventions with explanations.

**Adherent.** (1) Listed: metoprolol, torasemide, tolvaptan, rivaroxaban. (2) The dosage, frequency (“once daily”), and the fact that the regimen was maintained through hospitalization and at discharge are clearly documented in the Case Report. (3) It is explicitly stated that “This pharmacotherapeutic regimen remained unchanged throughout hospitalization and at discharge.”

**10. Follow-up and Outcomes**

- Clinician- and patient-assessed outcomes if available.
- Important follow-up diagnostic and other test results.
- Intervention adherence and tolerability. (How was this assessed?)
- Adverse and unanticipated events.

**Adherent.** (1) Outcome: symptom resolution at discharge and persistent NYHA class III at 3-month follow-up. (2)(4) The manuscript explicitly notes the absence of reported follow-up imaging/biomarker data and adverse events. (3) Formal assessment of long-term medication adherence and tolerability was not possible as the patient did not return for scheduled follow-up visits at our institution after discharge.

**11. Discussion**

- Strengths and limitations in your approach to this case.
- Discussion of the relevant medical literature.
- The rationale for your conclusions.
- The primary “take-away” lessons from this case report (without references) in a one paragraph conclusion.

**Adherent.** (1) Strengths: Multimodality approach; Limitations: Implicit (e.g., single case). (2) Extensive literature review on ApHCM with calcification (13 cases). (3) Rationale based on imaging-pathology-genetics integration. (4) The conclusion paragraph clearly states the takeaway lessons.

**12. Patient Perspective** – The patient should share their perspective on the treatment(s) they received.

**Adherent.** The patient's perspective was captured at the point of hospital discharge, with documentation that "the patient expressed satisfaction with the relief of symptoms." No further qualitative feedback was solicited during the subsequent follow-up period.

**13. Informed Consent** – The patient should give informed consent. (Provide if requested.)

**Adherent.** Ethics statement confirms written informed consent was obtained for publication.
